# Occurrence, function and evolutionary origins of ‘2A-like’ sequences in virus genomes

**DOI:** 10.1099/vir.0.83428-0

**Published:** 2008-04

**Authors:** Garry A. Luke, Pablo de Felipe, Alexander Lukashev, Susanna E. Kallioinen, Elizabeth A. Bruno, Martin D. Ryan

**Affiliations:** 1 Centre for Biomolecular Sciences, School of Biology, Biomolecular Sciences Building, University of St Andrews, North Haugh, St Andrews KY16 9ST, UK; 2 Institute of Poliomyelitis and Viral Encephalitides, Russian Academy of Medical Sciences, Moscow 142782, Russia

## Abstract

2A is an oligopeptide sequence mediating a ribosome ‘skipping’ effect, producing an apparent ‘cleavage’ of polyproteins. First identified and characterized in picornaviruses, ‘2A-like’ sequences are found in other mammalian viruses and a wide range of insect viruses. Databases were analysed using a motif conserved amongst 2A/2A-like sequences. The newly identified 2A-like sequences (30 aa) were inserted into a reporter polyprotein to determine their cleavage activity. Our analyses showed that these sequences fall into two categories. The majority mediated very high (complete) cleavage to separate proteins and a few sequences mediated cleavage with lower efficiency, generating appreciable levels of the uncleaved form. Phylogenetic analyses of 2A-like sequences and RNA-dependent RNA polymerases (RdRps) indicated multiple, independent, acquisitions of these sequences at different stages during virus evolution. Within a virus family, 2A sequences are (probably) homologous, but diverge due to other evolutionary pressures. Amongst different families, however, 2A/2A-like sequences appear to be homoplasic.

In entero- and rhinoviruses the capsid protein domain of the polyprotein (P1) is cleaved from the replicative protein domain (P2) by a proteinase (2A^pro^) that cleaves at its own N terminus (Fig. 1a[Fig f1]). In other genera of the family *Picornaviridae* (e.g. aphtho-, cardio-, erbo- and teschoviruses), the 2A protein produces an apparent co-translational ‘cleavage’ at its C terminus only requiring 2A plus the N-terminal proline of 2B (Ryan & Drew, 1994; Fig. 1a[Fig f1]). This is produced by a translational effect (ribosome ‘skipping’) rather than proteolysis (Ryan *et al.*, 1999; Donnelly *et al.*, 2001a; de Felipe *et al.*, 2003).

In the case of foot-and-mouth disease virus (FMDV), the 2A oligopeptide is post-translationally ‘trimmed’ from the C terminus of the upstream protein 1D by the virus-encoded 3C proteinase (3C^pro^), ‘delineating’ 2A as just 18 aa. Residues that were not critical, but enhanced the cleavage activity, mapped to a somewhat longer sequence, extending ∼30 aa upstream of the 2A/2B cleavage site (Donnelly *et al.*, 1997, 2001b). This length is consistent with our model of the cleavage mechanism, where 2A is proposed to interact with the exit tunnel of the ribosome to conformationally restrict the peptidyl-tRNA ester linkage, precluding it from nucleophilic attack by prolyl-tRNA in the A site of the ribosome (Ryan *et al.*, 1999; Donnelly *et al.*, 2001a).

A motif comprising the seven C-terminal residues of 2A and the N-terminal proline of protein 2B (underlined) is conserved (-DxExNPG^↓^P-, where ‘x’=any amino acid). With this motif, databases were analysed using pattinprot (Pôle BioIformatique Lyonnais) and psi-blast (NBCI; http://www.ncbi.nlm.nih.gov). The positions of 2A/2A-like sequences (2As) in a number of RNA viruses are shown in Fig. 1(b), the sequences are shown in Fig. 2 and GenBank accession numbers are listed in Supplementary Table S1 (available in JGV Online).

New 2A sequences found in the family *Picornaviridae* include: bovine rhinovirus 2 (BRV-2; Elizabeth Rieder, personal communication), Theiler-like virus of rats (T-LV; Ohsawa *et al.*, 2003), Saffold virus (SAF-V; Jones *et al.*, 2007), porcine teschoviruses (PTV; Zell *et al.*, 2001), Ljungan virus (LV; Lindberg & Johansson, 2002), Seneca valley virus (SVV; unpublished, GenBank accession no. DQ641257) and duck hepatitis virus (DHV-1 Kim *et al.*, 2006; Tseng *et al.*, 2007; New-DHV; Tseng & Tsai, 2007). 2As were also identified in newly characterized positive ssRNA insect viruses: (i) two in the iflaviruses Perina nuda picorna-like virus (PnPV; Wu *et al.*, 2002) and Ectropis obliqua picorna-like virus (EoPV; Wang *et al.*, 2004), (ii) a single 2A in the cripaviruses (family *Dicistroviridae*) acute bee paralysis virus (ABPV; Govan *et al.*, 2000), Kashmir bee virus (KBV; de Miranda *et al.*, 2004) and Israel acute paralysis virus (IAPV; Maori *et al.*, 2007) and (iii) a sequence in the betatetravirus (family *Tetraviridae*) *Euprosterna elaeasa* virus (EeV; Gorbalenya *et al.*, 2002) and three 2As in Providence virus (PrV; Pringle *et al.*, 2003; Fiona M. Pringle and L. Andrew Ball, personal communication).

We have previously reported the presence of active 2As in dsRNA type C rotavirus segment 6 (Donnelly *et al.*, 2001b). Re-analysing the databases revealed other 2As present within members of the family *Reoviridae*: (i) segment 5 of the human non-A, B, C rotavirus new adult diarrhea virus (ADRV-N; Yang *et al.*, 2004) and (ii) segment 5 of the insect *Operophtera brumata* cypovirus-18 (OpbuCPV-18; Graham *et al.*, 2006), *Lymantria dispar* cypovirus 1 (LdCPV-1; Rao *et al.*, 2003), *Bombyx mori* cypovirus 1 (BmCPV-1; Hagiwara *et al.*, 2001) and *Dendrolimus punctatus* cypovirus 1 (DpCPV-1; Zhao *et al.*, 2003). Furthermore, two 2As were found within the open reading frame (ORF) 1 of the dsRNA infectious myonecrosis virus of penaeid shrimp (IMNV; Poulos *et al.*, 2006; Nibert, 2007).

Sequences not completely matching the -DxExNPGP- motif were also identified: (i) three viruses belonging to the genus *Iflavirus*, Deformed wing virus (DWV; Lanzi *et al.*, 2006), Kakugo virus (KV; Fujiyuki *et al.*, 2004) and *Varroa destructor* virus-1 (VDV-1; Ongus *et al.*, 2004) and (ii) the unclassified picorna-like *Acyrthosiphon pisum* virus (APV; Van der Wilk *et al.*, 1997).

To study the activity of these 2As, plasmids were constructed to encode a single ORF consisting of green fluorescent protein (GFP), a longer (30 aa) version of 2A and *β*-glucuronidase (GUS; Donnelly *et al.*, 2001b). Oligonucleotide primers used are listed in Supplementary Table S2 (available in JGV Online). Rabbit reticulocyte lysate *in vitro* translation system (TnT T7 Quick Coupled Transcription/Translation System; Promega) was used to determine the cleavage activity of these new 2As. Proteins synthesized *de novo* were labelled with [^35^S]methionine (5 μCi, 185 kBq) and reactions were incubated at 30 °C for 90 min. Translation products were analysed by 10 % SDS-PAGE (Fig. 1c[Fig f1]) and the distribution of the radiolabel was quantified by using phosphorimaging. ‘Cleavage’ activities were calculated as described previously (Donnelly *et al.*, 2001b) and are the mean of three independent translation reactions.

In these *in vitro* systems, we typically observed three products: (i) low-level of [GFP-2A-GUS] uncleaved product, (ii) GUS and (iii) [GFP-2A] cleavage products. However, in picornavirus-infected cells, no proteins were detected that spanned the 2A/2B cleavage site (data not shown). Here, the longer versions of 2A more closely reflected the cleavage activities observed *in vivo* [∼99 % with sequences of the family *Picornaviridae* such as FMDV, equine rhinitis B virus 1 (ERBV-1), SAF-V and LV, Fig. 1(c)]. A variation from the consensus motif (-DVESNLGP-) reported in FMDV was found to be inactive (data not shown), consistent with analyses of site-directed mutants at this position (Donnelly *et al.*, 2001b). Interestingly, a rare substitution within this region (Ser→Pro; -DVEPNPGP-; Oem *et al.*, 2004; Carrillo *et al.*, 2005) cleaved highly efficiently (∼99 %; data not shown).

In insect iflaviruses, like the mammalian picornaviruses, 2A separates the capsid and the replicative protein domains (Fig. 1b[Fig f1]). Previous analysis of infectious flacherie virus (IFV; Isawa *et al.*, 1998) 2A showed lower cleavage (∼63 %; Donnelly *et al.*, 2001b). Again, the longer version was enough to enhance cleavage to ∼99 %, as with IFV and PnPV (Fig. 1c[Fig f1]). Interestingly, in the cases of PnPV and EoPV, both viruses have a second 2A between the structural VP2 and VP4 proteins (Fig. 1b[Fig f1]) that is also highly efficient (∼99 %; Fig. 1c[Fig f1]).

In members of the family *Dicistroviridae*, 2A occurs at the N-terminal region of the replicative protein ORF (Fig. 1b[Fig f1]). We have shown previously a high cleavage activity of the 18 aa 2As from *Drosophila* C virus (DCV, ∼95 %; Donnelly *et al.*, 2001b) and ABPV (∼94 %; Hughes 2003). The lower levels reported for cricket paralysis virus (CrPV, ∼88 %; Hughes 2003) were only marginally improved by extending the 2A sequence to 30 aa (∼90 %, Fig. 1c[Fig f1]).

Members of the insect family *Tetraviridae*, *Thosea asigna* virus (TaV), EeV and PrV (2A_3_) encode a 2A at the N terminus of the structural ORF (Fig. 1b[Fig f1]), which shows high cleavage activity (∼99 %, Fig. 1c[Fig f1]; Donnelly *et al.*, 2001b). PrV (2A_1_), in a non-structural ORF, cleaves very efficiently (∼99 %, Fig. 1c[Fig f1]), while PrV 2A_2_ has a somewhat lower activity (∼94 %; Fig. 1c[Fig f1]).

Two genera of the dsRNA family *Reoviridae* contain viruses with 2As in one of the segments encoding a non-structural protein (Fig. 1b[Fig f1]). In insect cypoviruses, a highly active 2A appears within segment 5 in BmCPV-1 and OpbuCPV-18 (∼99 %, Fig. 1c[Fig f1]). In rotaviruses, cleavage in segment 5 of human ADRV-N is highly efficient (∼97 %, Fig. 1c[Fig f1]), whereas in segment 6 of porcine and human type C rotaviruses it is lower (∼89 and ∼82 %, respectively; Fig. 1c[Fig f1]).

In type C rotaviruses, 2A links the ssRNA-binding protein NSP3 to dsRNA-binding protein (dsRBP). Rotavirus mRNAs do not bear poly(A) tails and NSP3 circularizes rotaviral mRNAs (Piron *et al.*, 1999; Jayaram *et al.*, 2004). The dsRBPs downstream of 2A sequester viral dsRNA (>11–16 nt, without apparent sequence specificity) from the cellular sensors of dsRNA, counteracting the activation of the cellular antiviral interferon system (Langland *et al.*, 1994). When segment 6 from the porcine C rotavirus was expressed, both *in vitro* and in COS-1 cells, similar to our *in vitro* analyses, three proteins were observed: a small amount of full-length [NSP3-2A-dsRBP] product and nearly equimolar amounts of [NSP3-2A] and the dsRBP cleavage products (Langland *et al.*, 1994). Furthermore, [NSP3-2A-dsRBP] was detected in infected cells and it was shown to bind dsRNA. It is noteworthy that NSP3 forms dimers, which may add a further level of complexity since NSP3 could form heterodimers with [NSP3-2A-dsRBP]. The incomplete cleavage produced by 2A allows type C rotaviruses to generate a complex array of products at relatively high levels. No other translational control mechanism can produce this outcome.

The members of the family *Totiviridae* are non-segmented dsRNA viruses. The N-terminal domain of the IMNV polyprotein ORF1 encodes non-structural proteins with two 2As (Fig. 1b[Fig f1]) that are highly active (∼99 %; Fig. 1c[Fig f1]). Interestingly, although segment 6 in group C rotavirus encodes a different protein to that of segment 5 in ADRV-N (NSP3 and NSP1, respectively), the protein downstream of 2A, a dsRBP, is the same in both cases. This dsRBP forms the N terminus of IMNV ORF1 followed by 2A_1_. In this case, therefore, the dsRBP is ‘cleaved’ from ORF1 as a [dsRBP-2A] protein.

The cleavage activity of 2As not completely matching the -DxExNPGP- motif was also determined. The iflaviruses VDV-1, KV and DWV contain the motif (-MDNPNPGP-) in the N-terminal region of their polyproteins. The VDV-1 2A was chosen for analysis and no cleavage activity was observed (data not shown). The unclassified picorna-like virus APV -DLESNPPP- sequence was modified (Pro→Gly, underlined) to closely resemble the consensus sequence (-DLESNPGP-). No cleavage activity was observed with either form of this sequence (data not shown).

Analyses of 2A-mediated cleavages suggest that they are of broadly two types. In most cases, very low levels of protein spanning the 2A tract are observed in our *in vitro* translation analyses. However, in CrPV, PrV-2A_2_ and type C rotaviruses there are appreciable levels (∼10 %) of uncleaved polyprotein *in vitro*. Currently, no data are available from CrPV- and PrV-infected cells.

Phylogenetic analyses of viruses containing 2As were performed to determine their evolutionary relationships by alignment of the RNA polymerases (RdRp) from 40 members of the picornavirus ‘supergroup’ and 19 other RNA viruses by using clustal w (Thompson *et al.*, 1994). All viruses with a functional 2A sequence were included in this analysis. Related viruses (without 2A) from the same families were included to produce a comprehensive phylogenetic tree (Fig. 3). RdRp sequences from the family *Tetraviridae* were, of necessity, excluded since major domains of the tetravirus RdRp are ‘shuffled’ in comparison with other RNA viruses (Gorbalenya *et al.*, 2002) and could not be aligned. Optimal alignments were obtained with the gap opening value set to 3 and gap extension set to 0.1. Phylogenetic trees were created using clustal_x 1.81 (neighbour-joining algorithm, Kimura substitution model) using the ‘exclude positions with gaps’ and ‘correct for multiple substitutions’ options. Phylogenetic trees were then visualized with NJPlot module. Phylogenetic relationships of the viruses were verified with previously published data. This analysis showed four major clusterings: two clusters with segmented dsRNA reoviruses (cypoviruses and rotaviruses), a single cluster with non-segmented dsRNA totiviruses and one comprising RdRps of all positive ssRNA viruses with separate branches formed by picornaviruses, iflaviruses and dicistroviruses (Fig. 3).

2As were aligned by using clustal_x 1.81 (Thompson *et al.*, 1997). Since 2A functions co-translationally (within the ribosome exit tunnel), we aligned these sequences such that no gaps were introduced by the algorithm (gap opening penalty=50, Fig. 2a[Fig f2]). It is apparent that 2A sequences from related viruses do not necessarily form clusters corresponding to those obtained using RdRp sequences, but are distributed throughout various branches of the tree (Fig. 2b–e[Fig f2]).

Capsid and replication proteins are separated in picornaviruses by three means: 3C^pro^, 2A^pro^ and the type of 2A that forms the subject of this paper. This region appears to be highly mutable – a recombinational hot-spot in entero- and aphthoviruses (Lukashev, 2005; Heath *et al.*, 2006). Since the latter form of 2A is present in many genera, either 2A has been acquired/lost on multiple occasions, or 2A was acquired at an early stage of evolution and subsequently replaced with a proteinase in the entero-, rhinovirus lineage (Fig. 3).

Whilst 2A appears to have been acquired at a relatively early stage in picornavirus evolution, the reverse seems to be the case in the dicistroviruses – assuming a single acquisition event in the branch comprising DCV, CrPV, *Solenopsis invicta* virus 1 (SINV-1), IAPV, KBV and ABPV. It appears that SINV-1 has lost 2A. Indeed, alignments show that SINV-1 has a large deletion of the N terminus of ORF1 (Valles *et al.*, 2004).

Similarly, acquisition of 2A appears to have occurred at a relatively late stage in the evolution of the members of the family *Reoviridae*. In cypoviruses, only the CPV-1 and -18 lineages possess 2As, while closely related viruses do not (e.g. LdCPV-14). In rotaviruses, a [2A-like/dsRBP] ‘module’ has been acquired by different RNA segments/proteins diverging into two forms: low cleavage (type C rotaviruses) and high cleavage (ADVR-N). Similarly, among the members of the family *Totiviridae*, only IMNV possesses a [2A-like/dsRBP] module (plus another downstream 2A).

A more complex pattern is observed in the iflaviruses. Here, analyses of both 2A and the polymerase sequences show IFV is much more distantly related to PnPV/EoPV (Figs 2c[Fig f2] and 3). Two explanations seem equally plausible: (i) an early acquisition accompanied by divergence of 2A (between IFV and PnPV/EoPV), acquisition of a second 2A in PnPV/EoPV and loss of 2A from the other lineages or, (ii) two independent acquisitions of 2A, one in IFV and another in the PnPV/EoPV lineage.

Our analysis suggests that 2As emerged independently at least six times amongst the RNA viruses analysed. Whilst some 2A sequences are clearly homologous, our data also strongly indicate homoplasy: a common function arising from multiple, independent, evolutionary origins – not surprising given their short length and the location of these sequences in known recombinational hot-spots.

## Supplementary Material

[Supplementary Tables]

## Figures and Tables

**Fig. 1. f1:**
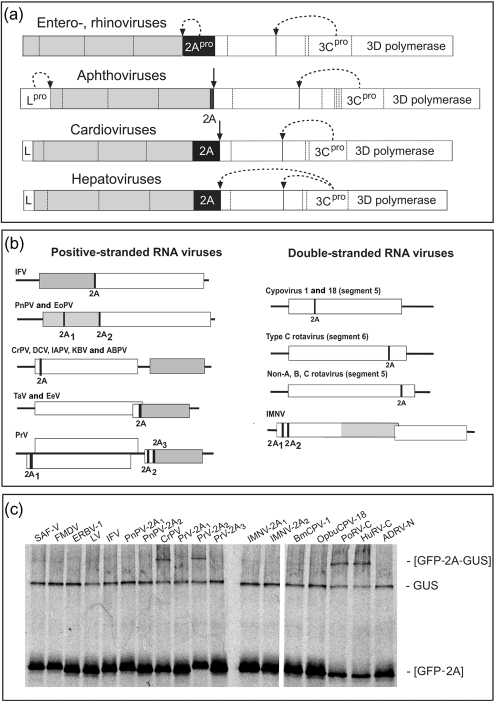
Polyprotein organization and translational analysis. (a) Polyprotein domains of four genera of the family *Picornaviridae* comprising capsid proteins (shaded areas), replicative proteins (open areas) and 2A sequences (black areas) are shown. Co-translational or ‘primary’ polyprotein cleavages mediated by virus-encoded proteinases are shown (curved dotted arrows). Subsequent (post-translational) cleavages within the polyprotein mediated by the 3C virus-encoded proteinase are indicated (vertical dotted lines). The co-translational primary cleavages at the C terminus of 2A (non-proteinase type) are shown (vertical solid arrows). (b) Genome organization of the positive ssRNA and dsRNA viruses containing 2A/2As. (c) Cleavage activity of 2As. Plasmids (0.1 μg) were used to programme rabbit reticulocyte lysates and the translation products were analysed by 10 % SDS-PAGE and quantified by phosphorimaging as described previously (Donnelly *et al.*, 2001b). Positions of the uncleaved [GFP-2A-GUS] and cleavage products GUS and [GFP-2A] are indicated.

**Fig. 2. f2:**
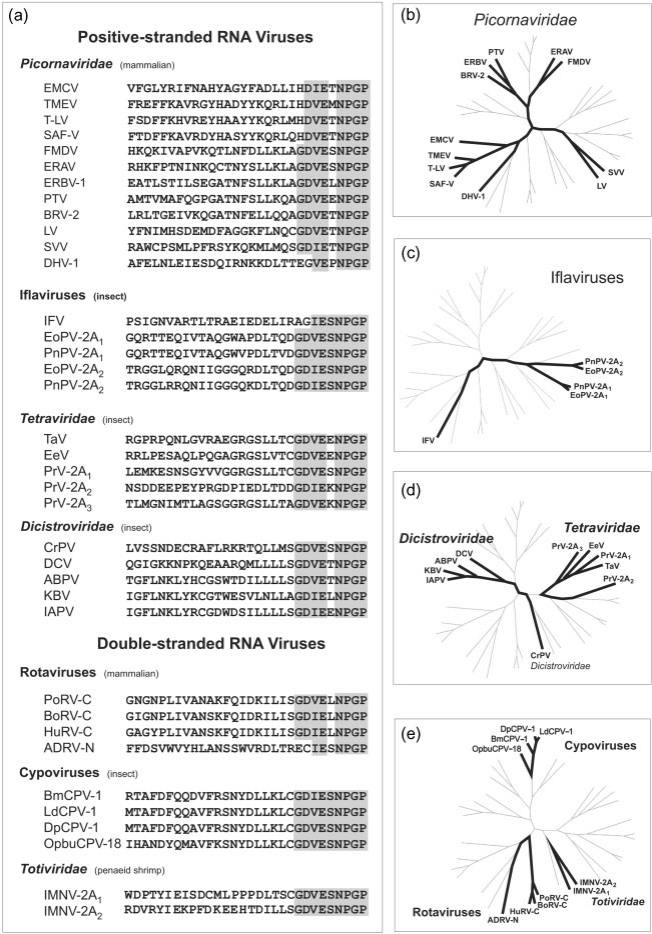
Phylogenetic analysis of 2A sequences. (a) Aligned 2A sequences are shown. (b–e) Phylogenetic trees have been reproduced, showing in each panel the clustering of particular virus groups (bold lines).

**Fig. 3. f3:**
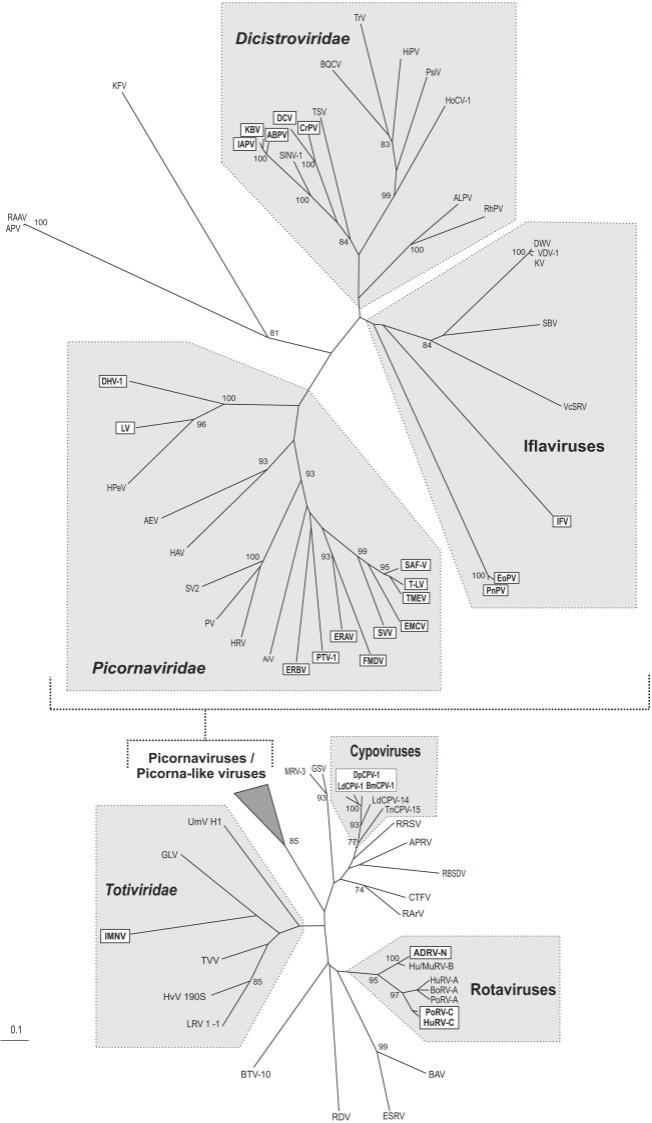
Phylogenetic analysis of RNA-dependent RNA polymerase (RdRp) sequences. Polymerase domains were aligned by using clustal_x and phylogenetic trees visualized by using NJPlot. Virus groups are indicated (shaded areas) with those viruses possessing 2As indicated in boxes. Virus names and sequence GenBank accession numbers are given in Supplementary Table S1 (available in JGV Online).
