# Supplementary material for: Occurrence, function and evolutionary origins of ‘2A-like’ sequences in virus genomes
Source: J Gen Virol. 2008 Apr;89(Pt 4):1036–42. doi: 10.1099/vir.0.83428-0 (PMC2885027; doi:10.1099/vir.0.83428-0)
Supplement: [Supplementary Tables] [file supp_89_4_1036__index.html]

 Occurrence, function and evolutionary origins of '2A-like' sequences in virus genomes -- Luke et al. 89 (4): 1036 Data Supplement - Supplementary Tables -- Journal of General Virology

## 

### Occurrence, function and evolutionary origins of ‘2A-like’ sequences in virus genomes, by G. A. Luke, P. de Felipe, A. Lukashev, S. E. Kallioinen, E. A. Bruno and M. D. Ryan

*Journal of General Virology* vol. **89**, part 4, pp. 1036 - 1042

**Supplementary Table S1.** RpRd and 2A/2As mentioned in this study

**Supplementary Table S2.** Oligonucleotide primers used throughout the study   
  
 [Single PDF file]  (162 KB)

  
  
