# Supplementary material for: Occurrence, function and evolutionary origins of ‘2A-like’ sequences in virus genomes
Source: J Gen Virol. 2008 Apr;89(Pt 4):1036–42. doi: 10.1099/vir.0.83428-0 (PMC2885027; doi:10.1099/vir.0.83428-0)
Supplement: [Supplementary Tables] [file supp_89_4_1036__1.pdf]

Supplementary Table S1. RpRd and 2A/2As mentioned in this study

Genus	Abbreviation	Full name	RdRp acc. no.	2A/2A-like acc. no.	Ref.
**Positive-stranded RNA viruses: family *Picornaviridae***					
Aphthovirus	FMDV	*Foot-and-mouth disease virus*	X00871	Idem	1
	ERAV	*Equine rhinitis A virus*	L43052	Idem	2
	BRV2	*Bovine rhinovirus 2*	-	E. Rieder, pers. com.	
Cardiovirus	EMCV	*Encephalomyocarditis virus*	M81861	Idem	3
	TMEV	*Theiler's murine encephalomyelitis virus*	X56019	Idem	4
	T-LV	*Theiler's-like virus of rats*	AB090161	Idem	5
	SAF-V	*Saffold virus*	EF165067	Idem	6
Erbovirus	ERBV	*Equine rhinitis B virus*	X96871	Idem	7
Teschovirus	PTV-1	*Porcine teschovirus 1*	AJ011380	Idem	8
New genus	DHV-1	*Duck hepatitis virus 1*	DQ219396	Idem	9
	N-DHV	*New duck hepatitis virus*	EF067924	Idem	10
New genus	SVV	*Seneca valley virus*	DQ641257	Idem	-
Parechovirus	LV	*Ljungan virus*	AF327921	Idem	11
	HPeV	*Human parechovirus 1*	S45208	–	12
Rhinovirus	HRV	*Human rhinovirus 1B*	D00239	–	13
Enterovirus	PV	*Human poliovirus 1*	V01149	–	14
Kobuvirus	AiV	*Aichi virus*	AB010145	–	15
Hepatovirus	HAV	*Hepatitis A virus*	M14707	–	16
	AEV	*Avian encephalomyelitis*	AJ225173	–	17
Sapelovirus	SV2	*Simian picornavirus 1*	AY064708	–	18
**FMDV strains with changes from the -DxExNPGP- canonical motif**					
**Strain/isolate**		**2A sequence**			
A10-61		-QLLNFDLLKLAGDVESN**LG** P-	X00429	Idem[a]	19
O/Ankara/TUR/377/10/02		-QLLNFDLLKLAGDVESN**LG** P-	–	DQ296523[a]	20
O/Ankara/TUR/643/12/99		-QLLNFDLLKLAGDVESN**LG** P-	–	DQ296516[a]	
O/Konya/TUR/512/10/99		-QLLNFDLLKLAGDVESN**LG** P-	–	DQ296515[a]	
O/Afyon/TUR/171/06/04		-QLLNFDLLKLAGDVESN**LG** P-	–	DQ296527[a]	
PanAsia O/SKR/2002		-QLLNFDLLKLAGDVE**P**NPG P-	AY312589	Idem	21
A1bayern iso41		-QLLNFDLLKLAGDVE**P**NPG P-	AY593759	Idem	22
**Positive-stranded RNA viruses: Iflaviruses (unassigned family)**					
Iflavirus	IFV	*Infectious flacherie virus*	AB000906	Idem	23
	PnPV	*Perina nuda picorna-like virus*	AF323747	Idem	24
	EoPV	*Ectropis obliqua picorna-like virus*	AY365064	Idem	25
	DWV	*Deformed wing virus*	AY292384	Idem[a]	26
	KV	*Kakugo virus*	AB070959	Idem[a]	27
	VDV-1	*Varroa destructor virus 1*	AY251269	Idem[a]	28
	SBV	*Sacbrood virus*	AF092924	–	29
	VcSRV	*Venturia canescens picorna-like virus*	AY534885	Undetermined	30
**Positive-stranded RNA viruses: family *Dicistroviridae***					
Cripavirus	CrPV	*Cricket paralysis virus*	AF218039	Idem	31
	DCV	*Drosophila C virus*	AF014388	Idem	32
	ABPV	*Acute bee paralysis virus*	AF150629	Idem	33
	KBV	*Kashmir bee virus*	AY275710	Idem	34
	IAPV	*Israel acute paralysis virus of bees*	EF219380	Idem	35
	SINV-1	*Solenopsis invicta virus 1*	AY634314	–	36
	TSV	*Taura syndrome virus*	AF277675	–	37
	BQCV	*Black queen cell virus*	AF183905	–	38
	TrV	*Triatoma virus*	AF178440	–	39
	HiPV	*Himetobi P virus*	AB017037	–	40
	PSIV	*Plautia stali intestine virus*	AB006531	–	41
	HoCV-1	*Homalodisca coagulata virus-1*	DQ288865	–	42
	RhPV	*Rhopalosiphum padi virus*	AF022937	–	43
	ALPV	*Aphid lethal paralysis virus*	AF536531	–	44

**Positive-stranded RNA viruses: unclassified picorna-like virus**					
	APV	*Acyrthosiphon pisum virus*	AF024514	Idem[a]	45
	RAAV	*Rosy apple aphid virus*	DQ286292	–	-
	KFV	*Kelp fly virus*	DQ112227	–	46
**Positive-stranded RNA viruses: family *Tetraviridae***					
Betatetravirus	TaV	*Thosea asigna virus*	AF282930	AF062037	47, 48
	EeV	*Euprosterna elaeasa virus*	AF461742	Idem	49
	PrV	*Providence virus*	–	AF548354 & A. Ball, pers. com.	50
**Segmented double-stranded RNA viruses: family *Reoviridae***					
Rotavirus	HuRV-A	*Human rotavirus A*	AB022765	–	-
	BoRV-A	*Bovine rotavirus A*	J04346	–	51
	PoRV-A	*Porcine rotavirus A*	M32805	–	52
	Hu/MuRV-B	*Human/murine rotavirus B, IDIR*	M97203	–	53
	HuRV-C	*Human rotavirus C*	AJ304859	AJ132203	54, 55
	BoRV-C	*Bovine rotavirus C*	–	L12390	56
	PoRV-C	*Porcine rotavirus C*	M74216	M69115	57, 58
	ADRV-N	*New adult diarrhoea virus / Adult diarrheal rotavirus str. J19*	DQ113897	DQ113901 =AY632079	59
Cypovirus	BmCPV-1	*Bombyx mori cypovirus 1*	AF323782	AB035732	60, 61
	DpCPV-1	*Dendrolimus punctatus cypovirus 1*	AY147187	AY163248	62
	LdCPV-1	*Lymantria dispar cypovirus 1*	AF389463	AF389466	63
	LdCPV-14	*Lymantria dispar cypovirus 14*	AF389452	–	63
	TnCPV-15	*Trichoplusia ni cypovirus 15*	AF291683	–	63
	OpbuCPV-18	*Operophtera brumata cypovirus 18*	–	DQ192245	64
Orthoreovirus	MRV-3	*Mammalian orthoreovirus-3*	M31058	–	65
Aquareovirus	GSV	*Golden shiner reovirus*	AF403399	–	66
Phytoreovirus	RDV	*Rice dwarf virus*	D10222	–	67
Coltivirus	CTFV	*Colorado tick fever virus*	AF133428	–	68
Seadornavirus	BAV	*Banna virus*	AF133430	–	69
Oryzavirus	RRSV	*Rice ragged stunt virus*	U66714	–	70
Fijivirus	RBSDV	*Rice black-streaked dwarf virus*	AJ294757	–	71
Mycoreovirus	RArV	*Rosellinia anti-rot virus*	AB102674	–	72
Dinovernavirus	APRV	*Aedes pseudoscutellaris reovirus*	DQ087277	–	73
Orbivirus	BTV-10	*Bluetongue virus-10*	X12819	–	74
Cardoreovirus	ESRV	*Eriocheir sinensis reovirus*	AY542965	–	75
**Non-segmented double-stranded RNA viruses: family *Totiviridae***					
Unclassified	IMNV	*Infectious myonecrosis virus*	AY570982	Idem	76
Giardiavirus	GLV	*Giardia lamblia virus*	L13218	–	77
Giardiavirus	TVV	Trichomonas vaginalis virus	U08999	–	78
Totivirus	UmV H1	*Ustilago maydis virus H1*	U01059	–	79
Totivirus	HvV 190S	*Helminthosporium victoriae virus 190S*	U41345	–	80
Leishmaniavirus	LRV 1-1	*Leishmania RNA virus 1-1*	M92355	–	81

[a]In these cases, the (reported) putative 2As are non-functional.

Supplementary Table S2. Oligonucleotide primers used throughout the study

Oligonucleotide Designation (sequence source)	Oligonuleotide (5′–3′)
Saff-rev (EF165067)	GCGCGCGGGCCCAGGGTTTGTTTCAACGTCATGTTGAAGTCTCTGTTTGTAATAAGAAGCATGATAGTCTCTA ACGGCTTTGAAAAAATCAGTGAATCTAGACCCGGACTTGTA
FMDVrev (X00871)	GCGCGCGGGCCCAGGGTTGGACTCGACGTCTCCCGCCAACTTGAGAAGGTCAAAATTCAAAGTCTGTTTCAC CGGTGCCACAATTTTCTGTTTGTGTCTAGACCCGGACTTGTA
ERBV1rev (X96871)	GCGCGCGGGCCCGGGGTTAAGTTCAACATCCCCTGCTAACTTCAACAAAGAAAAATTTGTGGCACCCTCAGA CAGAATGGTAGACAAAGTTGCTTCTCTAGACCCGGACTTGTA
LVrev (AF327921)	GCGCGCGGGCCCTGGGTTAGTTTCCACATCACCACATTGATTCAAAAATTTCCCCCCGGCAAAATCCATTTC ATCACTGTGCATTATATTAAAGTATCTAGACCCGGACTTGTA

IFVrev<br>(AB000906)	GCGCGCGGGCCCAGGATTTGATTCAATTCCTGCACGAATCAATTCATCCTCAATCTCCGCCCTCGTCAGAGT<br>CCGCGCGACATTACCAATTGAGGGTCTAGACCCGGACTTGTA
PnPVrev1<br>(AF323747)	GCGCGCGGGCCCGGGATTTGACTCAACATCTCCATCCACAGTCAAATCCGGAACCCACCCCTGGGCCGTAAC<br>TATCTGTTCAGTCGTCCTTTGTCCTCTAGACCCGGACTTGTA
PnPVrev2<br>(AF323747)	GCGCGCGGGCCCAGGATTCGACTCGATGTCACCATCTTGTGTCAAATCCTTCTGCCCACCACCAATAATATT<br>TTGCCGTCGTAAACCACCACGCGTTCTAGACCCGGACTTGTA
CrPVrev<br>(AF218039)	GCGCGCGGGCCCAGGATTAGATTCGACATCACCACTCATCAAAAGTTGCGTTCTCTTTCTCAAGAAGGCTCT<br>GCATTCATCATTACTTGAAACAAGTCTAGACCCGGACTTGTA
PROVrevA<br>(unpublished)	GCGCGCGGGCCCAGGGTTGGATTCAACGTCCCCACAAGTGAGAAGAGACCCCCGGCCACCGACTACGTAAC<br>CACTATTAGACTCCTTCATCTCCAATCTAGACCCGGACTTGTA
PROVrevB<br>(AF548354)	GCGCGCGGGCCCGGGGTTCTTCTCGATATCCCCATCATCTGTTAGATCTTCGATGGGGTCACCCCGCGGATAT<br>TCTGGCTCTTCGTCATCGCTGTTTCTAGACCCGGACTTGTA
PROVrevC<br>(AF548354)	GCGCGCGGGCCCAGGGTTCTTTTCAACATCGCCTGCGGTCAGCAAGCTTCCCCGACCACCTGACCCTGCAAGT<br>GTCATGATGTTCCCCATAAGCGTTCTAGACCCGGACTTGTA
IMNV1rev1<br>(AY570982)	GCGCGCGGGCCCAGGATTACTCTCAACGTCCCCGCACGATGTAAGGTCTGGAGGTGGCAGCATACAATCAGA<br>AATTTCAATGTAGGTTGGGTCCCATCTAGACCCGGACTTGTA
IMNVrev2<br>(AY570982)	GCGCGCGGGCCCGGGGTTTTCCTCAACATCACCAGATAACAGTATATCGGTATGTTCCTCTTTATCGAACGGC<br>TTTTCAATGTAACGAACATCCCTTCTAGACCCGGACTTGTA
PorCrev<br>(M69115)	GCGCGCGGGCCCAGGATTCAATTCAACATCTCCAGAAATTAAAATTTTGTCAATTTGGAATTTAGCATTTGC<br>AACAATCAATGGGTTACCATTACCTCTAGACCCGGACTTGTA
HumCrev<br>(AJ132203)	GCGCGCGGGCCCAGGATTTAATTCGATATCTCCGGAAATTAAAATTTTATCAATTTGAAATTTCGAATTTGC<br>GACAATCAGAGGGTAACCAGCACCTCTAGACCCGGACTTGTA
ADRV-Nrev<br>(DQ113901)	GCGCGCGGGCCCAGGGTTAGATTCAATGCATTCTCTAGTTAAATCTCGAACCCAAGAGCTGTTTGCCAAGTG<br>GTACACCCAAACCGAATCGAAGAATCTAGACCCGGACTTGTA
BmCPV1rev<br>(AB035732)	GCGGCGGGGCCCAGGATTAGACTCGATATCACCGCACAACTTTAGTAGGTCATAATTAGAGCGAAAAACGTC<br>CTGCTGGAAATCGAACGCTGTTCTTCTAGACCCGGACTTGTA
OpbuCPVrev<br>(DQ192245)	GCGCGCGGGCCCAGGATTTGATTCCACGTCCCCGCATAACTTCAGCAAATCATAATTTGATTTAAACACAGC<br>CATCTGATAATCATTAGCATGGATTCTAGACCCGGACTTGTA
FMDV-Pro-Leu<br>(PDFAR) (X00871)	GTGGTGGGGCCCCAGGTTGGACTCGACG
FMDV-Ser-Pro<br>(PDFAS) (X00871)	GTGGTGGGGCCCAGGGTTGGGCTCGACGTCTCC
VDV1rev<br>(AY251269)	GCGCGCGGGCCCAGGATTAGGATTGTCCATCTCAGGTTTAGCTGAAACAGGATTAGATAGCTGTAGCAAACT<br>AGTAACACACTCTAACTCGTATTCTCTAGACCCGGACTTGTA
APVfor1<br>(AF024514)	GCGCGCTCTAGATTAGAAACCAAAGCTTTTTATAACCGGCTTAATTATCCTATGCCTGAGGCATTACAAAAA<br>ATTATAGACTTAGAGTCAAATCCCCCTCCATTACGTCCTGTAGAAACC
APVfor2<br>(AF024514)	GCGCGCTCTAGATTAGAAACCAAAGCTTTTTATAACCGGCTTAATTATCCTATGCCTGAGGCATTACAAAAA<br>ATTATAGACTTAGAGTCAAATCCCGGGCCATTACGTCCTGTAGAAACC
T7for	TAATACGACTCACTATAGGG
pGUSrev	GCGCGCAATGCGATGCAATTTCCTCAT

References

1. **Forss, S., Strebel, K., Beck, E. & Schaller, H. (1984).** Nucleotide sequence and genome organization of foot-and-mouth disease virus. *Nucleic Acids Res* **12**, 6587–6601.
2. **Li, F., Browning, G. F., Studdert, M. J. & Crabb, B. S. (1996).** Equine rhinovirus 1 is more closely related to foot-and-mouth disease virus than to other picornaviruses. *Proc Natl Acad Sci U S A* **93**, 990–995.
3. **Duke, G. M., Hoffman, M. A. & Palmenberg, A.C. (1992).** Sequence and structural elements that contribute to efficient encephalomyocarditis virus RNA translation. *J Virol* **66**, 1602–1609.
4. **Law, K. M. & Brown, T. D. (1990).** The complete nucleotide sequence of the GDVII strain of Theiler's murine encephalomyelitis virus (TMEV). *Nucleic Acids Res* **18**, 6707–6708.
5. **Ohsawa, K., Watanabe, Y., Miyata, H. & Sato H. (2003).** Genetic analysis of a Theiler–like virus isolated from rats. *Comp Med* **53**, 191–196.
6. **Jones, M. S., Lukashov, V. V., Ganac, R. D. & Schnurr, D. P. (2007).** Discovery of a novel human picornavirus in a stool sample from a pediatric patient presenting with Fever of unknown origin. *J Clin Microbiol* **45**, 2144–2150.

7. **Wutz, G., Auer, H., Nowotny, N., Grosse, B., Skern, T. & Kuechler, E. (1996).** Equine rhinovirus serotypes 1 and 2: relationship to each other and to aphthoviruses and cardioviruses. *J Gen Virol* **77**, 1719–1730.
8. **Doherty, M., Todd, D., McFerran, N. & Hoey, E. M. (1999).** Sequence analysis of a porcine enterovirus serotype 1 isolate: relationships with other picornaviruses. *J Gen Virol* **80**, 1929–1941.
9. **Kim, M. C., Kwon, Y. K., Joh, S. J., Lindberg, A. M., Kwon, J. H., Kim, J. H. & Kim, S. J. (2006).** Molecular analysis of duck hepatitis virus type 1 reveals a novel lineage close to the genus *Parechovirus* in the family *Picornaviridae*. *J Gen Virol* **87**, 3307–3316.
10. **Tseng, C. H. & Tsai, H. J. (2007).** Molecular characterization of a new serotype of duck hepatitis virus. *Virus Res* **126**, 19–31.
11. **Lindberg, A. M. & Johansson, S. (2002).** Phylogenetic analysis of Ljungan virus and A–2 plaque virus, new members of the *Picornaviridae*. *Virus Res* **85**, 61–70.
12. **Hyypia, T., Horsnell, C., Maaronen, M., Khan, M., Kalkkinen, N., Auvinen, P., Kinnunen, L. & Stanway, G. (1992).** A distinct picornavirus group identified by sequence analysis. *Proc Natl Acad Sci U S A* **89**, 8847–8851.
13. **Hughes, P. J., North, C., Jellis, C. H., Minor, P. D. & Stanway, G. (1988).** The nucleotide sequence of human rhinovirus 1B: molecular relationships within the rhinovirus genus. *J Gen Virol* **69**, 49–58.
14. **Racaniello, V. R. & Baltimore, D. (1981).** Molecular cloning of poliovirus cDNA and determination of the complete nucleotide sequence of the viral genome. *Proc Natl Acad Sci U S A* **78**, 4887–4891.
15. **Yamashita, T., Sakae, K., Tsuzuki, H., Suzuki, Y., Ishikawa, N., Takeda, N., Miyamura, T. & Yamazaki, S. (1998).** Complete nucleotide sequence and genetic organization of Aichi virus, a distinct member of the *Picornaviridae* associated with acute gastroenteritis in humans. *J Virol* **72**, 8408–8412.
16. **Cohen, J. I., Ticehurst, J.R., Purcell, R. H., Buckler–White, A. & Baroudy, B. M. (1987).** Complete nucleotide sequence of wild–type hepatitis A virus: comparison with different strains of hepatitis A virus and other picornaviruses. *J Virol* **61**, 50–59.
17. **Marvil, P., Knowles, N. J., Mockett, A. P., Britton, P., Brown, T. D. & Cavanagh, D. (1999).** Avian encephalomyelitis virus is a picornavirus and is most closely related to hepatitis A virus. *J Gen Virol* **80**, 653–662.
18. **Oberste, M. S., Maher, K. & Pallansch, M. A. (2003).** Genomic evidence that simian virus 2 and six other simian picornaviruses represent a new genus in *Picornaviridae*. *Virology* **314**, 283–293.
19. **Carroll, A. R., Rowlands, D. J. & Clarke, B. E. (1984).** The complete nucleotide sequence of the RNA coding for the primary translation product of foot and mouth disease virus. *Nucleic Acids Res* **12**, 2461–2472.
20. **Klein, J., Parlak, U., Ozyoruk, F. & Christensen, L. S. (2006).** The molecular epidemiology of foot–and–mouth disease virus serotypes A and O from 1998 to 2004 in Turkey. *BMC Vet Res* **2**, 35.
21. **Oem, J. K., Lee, K. N., Cho, I. S., Kye, S. J., Park, J. H. & Joo, Y. S. (2004).** Comparison and analysis of the complete nucleotide sequence of foot–and–mouth disease viruses from animals in Korea and other PanAsia strains. *Virus Genes* **29**, 63–71.
22. **Carrillo, C., Tulman, E. R., Delhon, G., Lu, Z., Carreno, A., Vagnozzi, A., Kutish, G. F. & Rock, D. L. (2005).** Comparative Genomics of Foot–and–Mouth Disease Virus. *J Virol* **79**, 6487–6504.
23. **Isawa, H., Asano, S., Sahara, K., Iizuka, T. & Bando, H. (1998).** Analysis of genetic information of an insect picorna–like virus, infectious flacherie virus of silkworm: evidence for evolutionary relationships among insects, mammalian and plant picorna(–like) viruses. *Arch Virol* **143**, 127–143.
24. **Wu, C., Lo, C. F., Huang, C. J., Yu, H. T. & Wang, C. H. (2002).** The complete genome sequence of Perina nuda picorna–like virus, an insect–infecting RNA virus with a genome organization similar to that of the mammalian picornaviruses. *Virology* **294**, 312–323.
25. **Wang, X., Yhang, J., Lu, J., Yi, F., Liu, C. & Hu, Y. (2004).** Sequence analysis and genomic organization of a new insect picorna–like virus, Ectropis obliqua picorna–like virus, isolated from *Ectropis obliqua*. *J Gen Virol* **85**, 1145–1151.
26. **Lanzi, G., de Miranda, J. R., Boniotti, M. B., Cameron, C. E., Lavazza, A., Capucci, L., Camazine, S. M. & Rossi, C. (2006).** Molecular and biological characterization of deformed wing virus of honeybees (*Apis mellifera* L.). *J Virol* **80**, 4998–5009.
27. **Fujiyuki, T., Takeuchi, H., Ono, M., Ohka, S., Sasaki, T., Nomoto, A. & Kubo, T. (2004).** Novel insect picorna–like virus identified in the brains of aggressive worker honeybees. *J Virol* **78**, 1093–1100.
28. **Ongus, J. R., Peters, D., Bonmatin, J.–M., Bengsch, E., Vlak, J. M. & van Oers, M. M. (2004).** Complete sequence of a picorna–like virus of the genus *Iflavirus* replicating in the mite *Varroa destructor*. *J Gen Virol* **84**, 3747–3755.
29. **Ghosh, R. C., Ball, B. V., Willcocks, M. M. & Carter, M. J. (1999).** The nucleotide sequence of sacbrood virus of the honey bee: an insect picorna–like virus. *J Gen Virol* **80**, 1541–1549.
30. **Reineke, A. & Asgari, S. (2005).** Presence of a novel small RNA–containing virus in a laboratory culture of the endoparasitic wasp *Venturia canescens* (Hymenoptera: Ichneumonidae). *J Insect Physiol* **51**, 127–135.
31. **Wilson, J. E., Powell, M. J., Hoover, S. E. & Sarnow, P. (2000).** Naturally occurring dicistronic cricket paralysis virus RNA is regulated by two internal ribosome entry sites. *Mol Cell Biol* **20**, 4990–4999.

32. **Johnson, K. N. & Christian, P. D. (1998).** The novel genome organization of the insect picorna–like virus *Drosophila C* virus suggests this virus belongs to a previously undescribed virus family. *J Gen Virol* **79**, 191–203.
33. **Govan, V. A., Leat, N., Allsopp, M. & Davison, S. (2000).** Analysis of the complete genome sequence of acute bee paralysis virus shows that it belongs to the novel group of insect–infecting RNA viruses. *Virology* **277**, 457–463.
34. **de Miranda, J. R., Drebot, M., Tyler, S., Shen, M., Cameron, C. E., Stoltz, D. B. & Camazine, S. M. (2004).** Complete nucleotide sequence of Kashmir bee virus and comparison with acute bee paralysis virus. *J Gen Virol* **85**, 2263–2270.
35. **Maori, E., Tanne, E. & Sela, I. (2007).** Reciprocal sequence exchange between non–retro viruses and hosts leading to the appearance of new host phenotypes. *Virology* **362**, 342–349.
36. **Valles, S. M., Strong, C. A., Dang, P. M., Hunter, W. B., Pereira, R. M., Oi, D. H., Shapiro, A. M. & Williams, D. F. (2004).** A picorna–like virus from the red imported fire ant, *Solenopsis invicta*: initial discovery, genome sequence, and characterization. *Virology* **328**, 151–157.
37. **Mari, J., Poulos, B. T., Lightner, D. V. & Bonami, J. R. (2002).** Shrimp Taura syndrome virus: genomic characterization and similarity with members of the genus *Cricket paralysis–like* viruses. *J Gen Virol* **83**, 915–926.
38. **Leat, N., Ball, B., Govan, V. & Davison, S. (2000).** Analysis of the complete genome sequence of black queen–cell virus, a picorna–like virus of honey bees. *J Gen Virol* **81**, 2111–2119.
39. **Czibener, C., la Torre, J. L., Muscio, O. A., Ugalde, R. A. & Scodeller, E. A. (2000).** Nucleotide sequence analysis of Triatoma virus shows that it is a member of a novel group of insect RNA viruses. *J Gen Virol* **81**, 1149–1154.
40. **Nakashima, N., Sasaki, J. & Toriyama, S. (1999).** Determining the nucleotide sequence and capsid–coding region of Himetobi P virus: a member of a novel group of RNA viruses that infect insects. *Arch Virol* **144**, 2051–2058.
41. **Sasaki, J., Nakashima, N., Saito, H. & Noda, H. (1998).** An insect picorna–like virus, *Plautia stali* intestine virus, has genes of capsid proteins in the 3' part of the genome. *Virology* **244**, 50–58.
42. **Hunnicutt, L. E; Hunter, W. B; Cave, R. D; Powell, C. A. & Mozoruk, J. J. (2006).** Genome sequence and molecular characterization of *Homalodisca coagulata virus–1*, a novel virus discovered in the glassy–winged sharpshooter (Hemiptera: Cicadellidae). *Virology* **350**, 67–78.
43. **Moon, J. S., Domier, L. L., McCoppin, N. K., D'Arcy, D. J. & Jin, H. (1998).** Nucleotide sequence analysis shows that *Rhopalosiphum padi* virus is a member of a novel group of insect–infecting RNA viruses. *Virology* **243**, 54–65.
44. **van Munster, M., Dullermans, A. M., Verbeek, M., van der Heuvel, J. F. J. M., Clérivet, A & van der Wilk, F. (2002).** Sequence analysis and genomic organization of Aphid lethal paralysis virus: a new member of the family *Dicistroviridae*. *J Gen Virol* **83**, 3131–3138.
45. **Van der Wilk, F., Dullemans, A. M., Verbeek, M. & van den Heuvel, J. F. J. M. (1997).** Nucleotide sequence and genomic organization of Acyrthosiphon Pisum Virus. *Virology* **238**, 353–362.
46. **Hartley, C. J., Greenwood, D. R., Gilbert, R. J., Masoumi, A., Gordon, K. H., Hanzlik, T.N., Fry, E. E., Stuart, D. I. & Scotti, P. D. (2005).** Kelp fly virus: a novel group of insect picorna–like viruses as defined by genome sequence analysis and a distinctive virion structure. *J Virol* **79**, 13385–13398.
47. **Pringle, F. M., Gordon, K. H. J., Hanzlik, T. N., Kalmakoff, J., Scotti, P. D. & Ward, V. K. (1999).** A novel capsid expression strategy for *Thosea asigna* virus (*Tetraviridae*). *J Gen Virol* **80**, 1855–1863.
48. **Pringle, F. M., Kalmakoff, J. & Ward, V. K. (2001).** Analysis of the capsid processing strategy of *Thosea asigna* virus using baculovirus expression of virus–like particles. *J Gen Virol* **82**, 259–266.
49. **Gorbalenya, A. E., Pringle, F. M., Zeddam, J. L., Luke, B. T., Cameron, C.E., Kalmakoff, J., Hanzlik, T.N., Gordon, K. H. J. & Ward, V. K. (2002).** The palm subdomain–based active site is internally permuted in viral RNA–dependent RNA polymerases of an ancient lineage. *J Mol Biol* **324**, 47–62.
50. **Pringle, F. M., Johnson, K. N., Goodman, C. L., McIntosh, A. H. & Ball, L. A. (2003).** Providence virus: a new member of the *Tetraviridae* that infects cultured insect cells. *Virology* **306**, 359–370.
51. **Cohen, J., Charpilienne, A., Chilmonczyk, S. & Estes, M. K. (1989).** Nucleotide sequence of bovine rotavirus gene 1 and expression of the gene product in baculovirus. *Virology* **171**, 131–140.
52. **Fukuhara, N., Nishikawa, K., Gorziglia, M. & Kapikian, A. Z. (1989).** Nucleotide sequence of gene segment 1 of a porcine rotavirus strain *Virology* **173**, 743–749.
53. **Eiden, J. J. & Hirshon, C. (1993).** Sequence analysis of group B rotavirus gene 1 and definition of a rotavirus–specific sequence motif within the RNA polymerase gene. *Virology* **192**, 154–160.
54. **Chen, Z., Lambden, P. R., Lau, J., Caul, E. O. & Clarke, I. N. (2002).** Human group C rotavirus: completion of the genome sequence and gene coding assignments of a non–cultivatable rotavirus. *Virus Res* **83**, 179–187.
55. **James, V. L., Lambden, P. R., Deng, Y., Caul, E. O. & Clarke, I. N. (1999).** Molecular characterization of human group C rotavirus genes 6, 7 and 9. *J Gen Virol* **80**, 3181–3187.

56. **Jiang, B., Tsunemitsu, H., Gentsch, J. R., Saif, L. J. & Glass, R. I. (1993).** Nucleotide sequences of genes 6 and 10 of a bovine group C rotavirus. *Nucleic Acids Res* **21**, 2250.
57. **Bremont, M., Juste–Lesage, P., Chabanne–Vautherot, D., Charpilienne, A. & Cohen, J. (1992).** Sequences of the four larger proteins of a porcine group C rotavirus and comparison with the equivalent group A rotavirus proteins. *Virology* **186**, 684–692.
58. **Qian, Y. A., Jiang, B. M., Saif, L. J., Kang, S. Y., Ojeh, C. K. & Green, K. Y. (1991).** Molecular analysis of the gene 6 from a porcine group C rotavirus that encodes the NS34 equivalent of group A rotaviruses *Virology* **184**, 752–757.
59. **Yang, H., Makeyev, E. V., Kang, Z., Ji, S., Bamford, D. H. & van Dijk, A. A. (2004).** Cloning and sequence analysis of dsRNA segments 5, 6 and 7 of a novel non–group A, B, C adult rotavirus that caused an outbreak of gastroenteritis in China. *Virus Res* **106**, 15–26.
60. **Hagiwara, K., Rao, S., Scott, S. W. & Carner, G. R. (2002).** Nucleotide sequences of segments 1, 3 and 4 of the genome of Bombyx mori cypovirus 1 encoding putative capsid proteins VP1, VP3 and VP4, respectively *J Gen Virol* **83**, 1477–1482.
61. **Hagiwara, K., Kobayashi, J., Tomita, M. & Yoshimura, T. (2001).** Nucleotide sequence of genome segment 5 from *Bombyx mori* cypovirus 1. *Arch Virol* **146**, 181–187.
62. **Zhao, S. L., Liang, C. Y., Hong, J. J. & Peng, H. Y. (2003).** Genomic sequence analysis of segments 1 to 6 of Dendrolimus punctatus cytoplasmic polyhedrosis virus. *Arch Virol* **148**, 1357–1368.
63. **Rao, S., Carner, G. R., Scott, S. W., Omura, T. & Hagiwara, K. (2003).** Comparison of the amino acid sequences of RNA–dependent RNA polymerases of cypoviruses in the family *Reoviridae*. *Arch Virol* **148**, 209–219.
64. **Graham, R. I., Rao, S., Possee, R. D., Sait, S. M., Mertens, P. P. C. & Hails, R. S. (2006).** Detection and characterization of three novel species of reovirus (Reoviridae), isolated from geographically separate populations of the winter moth *Operophtera brumata* (Lepidoptera: Geometridae) on Orkney. *J Invertebr Pathol* **91**, 79–87.
65. **Wiener, J. R. & Joklik, W. K. (1989).** The sequences of the reovirus serotype 1, 2, and 3 L1 genome segments and analysis of the mode of divergence of the reovirus serotypes. *Virology* **169**, 194–203.
66. **Attoui, H., Fang, Q., Jaafar, F. M., Cantaloube, J. F., Biagini, P., De Micco, P. & De Lamballerie, X. (2002).** Common evolutionary origin of aquareoviruses and orthoreoviruses revealed by genome characterization of Golden shiner reovirus, Grass carp reovirus, Striped bass reovirus and golden ide reovirus (genus *Aquareovirus*, family *Reoviridae*). *J Gen Virol* **83**, 1941–1951.
67. **Suzuki, N., Tanimura, M., Watanabe, Y., Kusano, T., Kitagawa, Y., Suda, N., Kudo, H., Uyeda, I. & Shikata, E. (1992).** Molecular analysis of rice dwarf phytoreovirus segment S1: interviral homology of the putative RNA–dependent RNA polymerase between plant– and animal–infecting reoviruses. *Virology* **190**, 240–247.
68. **Attoui, H ., Billoir, F., Biagini, P., Cantaloube, J. F., de Chesse, R., de Micco, P. & de Lamballerie, X. (2000).** Sequence determination and analysis of the full–length genome of colorado tick fever virus, the type species of genus *Coltivirus* (Family *Reoviridae*). *Biochem Biophys Res* Commun **273**, 1121–1125.
69. **Attoui, H., Billoir, F., Biagini, P., de Micco, P. & de Lamballerie, X. (2000).** Complete sequence determination and genetic analysis of Banna virus and Kadipiro virus: proposal for assignment to a new genus (*Seadornavirus*) within the family *Reoviridae*. *J Gen Virol* **81**, 1507–1515.
70. **Upadhyaya, N. M., Ramm, K., Gellatly, J. A., Li, Z., Kositratana, W. & Waterhouse, P. M. (1998).** Rice ragged stunt oryzavirus genome segment S4 could encode an RNA dependent RNA polymerase and a second protein of unknown function. *Arch Virol* **143**, 1815–1822.
71. **Zhang, H. M., Chen, J. P. & Adams, M. J. (2001).** Molecular characterisation of segments 1 to 6 of Rice black–streaked dwarf virus from China provides the complete genome. *Arch Virol* **146**, 2331–2339.
72. **Wei, C. Z., Osaki, H., Iwanami, T., Matsumoto, N. & Ohtsu, Y. (2004).** Complete nucleotide sequences of genome segments 1 and 3 of Rosellinia anti–rot virus in the family *Reoviridae*. *Arch Virol* **149**, 773–777.
73. **Attoui, H., Mohd Jaafar, F., Belhouchet, M., Biagini, P., Cantaloube, J. F., de Micco, P. & de Lamballerie, X. (2005).** Expansion of family Reoviridae to include nine–segmented dsRNA viruses: Isolation and characterization of a new virus designated aedes pseudoscutellaris reovirus assigned to a proposed genus (Dinovernavirus). *Virology* **343**, 212–223.
74. **Roy, P., Fukusho, A., Ritter, G. D. & Lyon, D. (1988).** Evidence for genetic relationship between RNA and DNA viruses from the sequence homology of a putative polymerase gene of bluetongue virus with that of vaccinia virus: conservation of RNA polymerase genes from diverse species. *Nucleic Acids Res* **16**, 11759–11767.
75. **Zhang, S., Shi, Z., Zhang, J. & Bonami, J. R. (2004).** Purification and characterization of a new reovirus from the Chinese mitten crab, *Eriocheir sinensis*. *J Fish Dis* **27**, 687–692.
76. **Poulos, B. T., Tang, K. F., Pantoja, C. R., Bonami, J. R. & Lightner, D. V. (2006).** Purification and characterization of infectious myonecrosis virus of penaeid shrimp. *J Gen Virol* **87**, 987–996.

77. **Wang, A.L., Yang, H. M., Shen, K. A. & Wang, C. C. (1993).** Giardiavirus double–stranded RNA genome encodes a capsid polypeptide and a gag–pol–like fusion protein by a translation frameshift. *Proc Natl Acad Sci U S A* **90**, 8595–8599.
78. **Tai, J. H. & Ip, C. F. (1995).** The cDNA sequence of *Trichomonas vaginalis* virus–T1 double–stranded RNA *Virology* **206**, 773–776.
79. **Bruenn, J. A. (1993).** A closely related group of RNA–dependent RNA polymerases from double–stranded RNA viruses. *Nucleic Acids Res* **21**, 5667–5669.
80. **Huang, S. & Ghabrial, S. A. (1996).** Organization and expression of the double–stranded RNA genome of *Helminthosporium victoriae* 190S virus, a totivirus infecting a plant pathogenic filamentous fungus. *Proc Natl Acad Sci U S A* **93**, 12541–12546.
81. **Stuart, K. D., Weeks, R., Guilbride, L. & Myler, P. J. (1992).** Molecular organization of *Leishmania* RNA virus 1. *Proc Natl Acad Sci U S A* **89**, 8596–8600.
